# Adherence to Multidisciplinary Tumor Board Recommendations in Patients with Multiple Myeloma

**DOI:** 10.3390/cancers17081297

**Published:** 2025-04-11

**Authors:** Valérie Gennheimer, Dilara Akhoundova, Michèle Hoffmann, Barbara Jeker, Yara Banz, Ulrike Bacher, Thomas Pabst

**Affiliations:** 1 Department of Medical Oncology, Inselspital, Bern University Hospital, University of Bern, 3010 Bern, Switzerland; valerie.gennheimer@students.unibe.ch (V.G.); dilara.akhoundovasanoyan@insel.ch (D.A.); michele.hoffmann@insel.ch (M.H.); barbara.jeker@insel.ch (B.J.); 2 Institute of Pathology, University of Bern, 3010 Bern, Switzerland; yara.banz@unibe.ch; 3 Department of Hematology, Inselspital, Bern University Hospital, University of Bern, 3010 Bern, Switzerland; veraulrike.bacher@insel.ch

**Keywords:** multiple myeloma (MM), multidisciplinary tumor boards (MTB), adherence, implementation

## Abstract

Background: Patient management following a multidisciplinary tumor board (MTB) recommendation has become standard of care in oncology and aims to ensure optimization and personalization of patient care. To assess the impact of MTB recommendations in clinical practice, adherence to the recommended procedures needs to be evaluated. Within this retrospective case series, we examined adherence rates to recommendations formulated at multidisciplinary myeloma tumor boards (MMTB) held at our institution. Specifically, we analyzed how often recommendations involving diagnostic procedures, therapies, and enrollment into clinical trials recommended by the MMTB were implemented. In addition, factors leading to non-adherence were evaluated. Methods: We reviewed all consecutive patient cases discussed at MMTBs held at the University Hospital of Bern, Switzerland, between 1 January and 31 December 2023. Adherence was assessed by systematically comparing all available clinical records with the recommendations formulated at the MMTBs. Results: In total, 218 patients were included in the study. Of all MMTB recommendations, 86% (n = 251) of all MMTB recommendations were followed. Of these, 84% (n = 244) were followed with complete adherence and 2% (n = 7) incompletely. All cases of non-implementation of MMTB recommendations concerning diagnostics or therapy were clinically justified. The main reason for non-adherence was patient decision. Other reasons included lack of cost coverage and relevant changes in the clinical scenario, including patient’s death. In total, 36% (n = 104) of MMTB recommendations included clinical trial enrollment. However, study enrollment occurred only in 32% (n = 33) of the 104 cases. In 41% (n = 29) of the cases, justification for non-enrollment was documented in the clinical records. The most frequent reasons were patient decision, unmet inclusion criteria, delays in recruitment, lack of reimbursement, and changes in the clinical scenario. Conclusions: Our study showed an overall high level of adherence to MMTB recommendations for diagnostic procedures and therapy. However, only one third of recommendations for clinical trial enrollment were implemented, frequently due to patient decisions. Our results highlight the relevance of regular assessments of adherence rates to MTB recommendations and suggest that considering patient preferences in MTB discussions might minimize deviations.

## Simple Summary

Multidisciplinary tumor boards (MTB) have become the standard of care in oncology; they aim to ensure high-quality and personalized patient care. Previous reports suggest that adherence to MTB recommendations leads to improved clinical outcomes, including treatment responses and progression-free and overall survival, as well as increasing patient recruitment into clinical trials. Within this study, we analyzed adherence rates to recommendations formulated at multidisciplinary myeloma tumor boards (MMTB) held between January and December 2023 at the University Hospital of Bern, Switzerland. Specifically, we analyzed how often recommendations regarding diagnostics, therapy, and trial enrollment proposed by the MMTB were indeed followed. In addition, factors leading to non-adherence were evaluated. Overall, we found a high level of adherence to diagnostic and therapeutic recommendations. However, only approximately one third of recommendations for study enrollment were implemented.

## 1. Introduction

In Switzerland, approximately 730 patients are diagnosed with multiple myeloma (MM) every year, and increasing incidence is reported [1,2,3]. Advances in treatment have significantly improved patient outcomes, including overall survival (OS) rate, for MM patients over the past few decades [2,4,5,6]. The broad spectrum of currently available diagnostic approaches and treatment options, however, make choosing the optimal diagnostic and therapeutic algorithm considerably more challenging [7,8,9]. This holds true both when myeloma is diagnosed and during its clinical course. In order to ensure that every patient receives optimal and personalized management, multidisciplinary tumor boards (MTB) have been established as a modern standard in oncology care [7,10]. The aim of this collaboration between medical specialists from different disciplines is to formulate recommendations that are both patient-oriented and supported by solid scientific rationale [11]. Previous studies have shown that cancer patients benefit from this multidisciplinary approach [11,12]. Specifically, the adherence to MTB recommendations has led to improved treatment response rates, progression-free survival (PFS), or longer OS [11,12]. These data highlight the positive impact of implementing MTB decisions on patients’ outcomes [11].

Improved patient recruitment into clinical trials is another advantage of MTBs [7,10,13,14]. International guidelines, including the National Comprehensive Cancer Network (NCCN), recommend enrollment into clinical trials as a valuable treatment option for patients diagnosed with cancer [14,15]. Several studies have suggested that participation in clinical trials improves patient outcomes [16]. However, a recent systematic review and meta-analysis by Iskander et al. questioned this often-implied benefit for clinical trial participants [16,17]. Nonetheless, clinical trials remain essential to further advance oncological treatments and enable approval of novel drugs and treatment approaches. Therefore, large national and international professional oncology associations recommend treating a significant proportion of oncology patients in clinical studies [14,18,19].

According to the certification program of the German Cancer Society (Deutsche Krebsgesellschaft, DKG), therapy recommendations of the MTBs are considered binding [20]. Therefore, DKG-certified cancer centers must monitor the implementation of the MTB recommendations [20,21]. Deviations should be documented and underlying reasons evaluated in order to prevent deviations [20]. Regular controls of adherence to MTB recommendations are essential for ensuring high-quality management of cancer patients [21,22].

Despite the central role of MTBs in current management of cancer treatment and the growing need for systematic monitoring of adherence to MTB recommendations, only two studies that specifically addressed adherence to multidisciplinary myeloma tumor boards (MMTB) could be identified [7,10]. These two studies, conducted at the same center, showed adherence rates to MMTB recommendations of over 90% [7]. Reasons for non-compliance with recommendations were all well-founded and mostly related to patient decisions [7,10]. The study results of Engelhardt et al. also showed that trial enrolment was implemented in only 53.2% of the cases. Reasons for non-inclusion were unmet inclusion criteria or decisions made by patients and relatives [10].

Overall, there is a large discrepancy in the literature regarding adherence to MTB recommendations. According to Christ et al., the general adherence rate is between 58.2% and 89.6% [23].

The aim of this retrospective case series was to examine adherence to multidisciplinary myeloma tumor board (MMTB) recommendations at the University Hospital of Bern (Switzerland). Specifically, we analyzed the implementation rate for recommendations concerning diagnostics, therapy and trial enrolment. Moreover, we evaluated reasons and factors leading to non-adherence to formulated recommendations.

## 2. Materials and Methods

### 2.1. Data Collection

All consecutive recommendations formulated at weekly MMTBs at the University Hospital of Bern, Switzerland, between 1 January 2023 and 31 December 2023 were retrospectively reviewed to assess their implementation. Patients treated at external institutions and referred for MMTB discussion were presented via video conference by their treating physicians. The University Hospital Bern is a center certified by the German Cancer Society (Deutsche Krebsgesellschaft, DKG). Each week, these MMTBs discussed the management of patients diagnosed with MM, smoldering myeloma, monoclonal gammopathies of undetermined significance and amyloidosis. Following internal guidelines, patients are regularly presented at the MMTB at diagnosis, before ASCT and maintenance indication, as well as at relapse/disease progression. The multidisciplinary team consisted of oncologists, hematologists, radiologists, radiation oncologists, and pathologists specialized in plasma cell malignancies. Depending on the clinical question and the patient’s need, additional specialist disciplines, such as cardiologists, dermatologists, hematological cytogeneticist, nuclear medicine physicians, orthopedists, nephrologists, and neurologists, were involved.

Since MM patients frequently required several treatment lines, many of these patients were presented at MMTBs in several occasions during the one-year period [24]. Each discussion in the MMTB was counted and analyzed as a single case.

All recommendations of the MMTBs, which focused specifically on diagnostics as well as therapy and trial enrolment recommendations, were recorded on the respective registration form. The MMTB recommendations to be reviewed could, therefore, be found in these records, stored in the relevant patient files in the hospital program EPIC. The data used for the adherence analysis were extracted mainly from this program. Additionally, the Marcell and Onkostar databases (tumor documentation system) were used in individual cases.

For the evaluation, all data were collected manually in an Excel spreadsheet. The following information was captured for each patient: age at initial diagnosis and board presentation, gender, diagnosis, stage of disease, status of disease, MMTB recommendations, diagnostics and therapy measures implemented following the MMTB, reasons for non-adherence.

### 2.2. Assessment and Analysis of MMTB Adherence

By comparing all available documentation data with the recommendations of the MMTBs, adherence could be evaluated. The following recommendations were analyzed: diagnostic procedures, recommendations involving systemic therapies (chemotherapy and/or immunotherapy, maintenance therapy, high-dose chemotherapy (HDCT) with autologous stem cell transplantation (ASCT), tandem ASCT, CAR-T cell therapy, bispecific antibodies), radiotherapy, watch and wait strategy, and other treatment proposals. The diagnostic procedures included the recommendations for further oncologically relevant clarifications (e.g., tumor biopsy, bone marrow biopsy, Fluorescence in situ hybridization, imaging, PET-CT, CT, MRI). If a cardio-oncological assessment (e.g., transthoracic echocardiography, electrocardiogram) and a pulmonary function test were recommended prior to HDCT with ASCT, only whether or not HDCT with ASCT was carried out was verified. Recommendations for bisphosphonate therapies were not assessed due to a loack of clarity in clinical records regarding implementation time-point.

Adherence to MMTB recommendations was rated as: completely adhered, incompletely adhered, or not adhered to. A MMTB recommendation was considered completely adhered to if all the recommended diagnostic and therapy procedures were implemented. In case of partial deviation from recommendations concerning diagnostic procedures with full implementation of therapy recommendation, the recommendation was rated as incompletely adhered to. Any non-implemented recommendations concerning therapy were rated as non-adhered to.

If recommendations were not followed, we assessed whether deviations were justified. Causes of deviations were classified as either plausible (e.g., due to patient clinical deterioration) or implausible for cases lacking justification.

The frequency with which patients were proposed for inclusion in a study was dealt with separately. If the MMTB recommended participation in a study, the reports and the hospital program EPIC were checked to see if inclusion in a study had been documented. If no study inclusion was recorded, the documentation was searched for reasons and, if available, collected in Excel.

Inclusion of patients in the recommended studies by the end of November 2024 was considered adherent. If several studies were suggested at a MMTB, participation in one study was sufficient to be considered as adherence. Information regarding study enrollment for patients managed outside our institution was not available.

### 2.3. Statistical Analyses

The data were analyzed using Microsoft Office Excel 2023. Descriptive statistics were used to analyze categorical and continuous variables of interest. The variables assessed were frequency of case presentations and MMTB recommendations, number of patients, patient basal characteristics, adherence to the MMTB recommendations, and the reasons for deviations.

## 3. Results

### 3.1. Multidisciplinary Myeloma Tumor Boards

Between 1 January 2023 and 31 December 2023, 218 patients were discussed in 290 MMTB cases. All patients and MMTBs were included in the analysis. The median number of MMTB per patient during the evaluation period was one, with a range of 1–4. In total, 74% (n = 162) of patients presented once, 20% (n = 43) twice, 5% (n = 10) three times, and 3 patients (1%) presented four times. The median number of cases discussed per MMTB meeting was 6, with a range of 1–15.

In addition, Table 1 shows the recommendations made by the MMTBs. Most MMTBs included several recommendations. Chemotherapy and/or immunotherapy were recommended in 37% (n = 108) of MMTBs. Regarding the frequency of the other therapy proposals, see Table 1. In total, further oncological evaluations were recommended in 27% (n = 77) of MMTBs. Of these, 6% (n = 16) only contained diagnostic recommendations, while 21% (n = 61) included both therapeutic and diagnostic recommendations. In 36% (n = 104) of MMTB cases, participation in a clinical study was recommended.

### 3.2. Patient Basal Characteristics

Patient basal characteristics are summarized in Table 2. Of the 218 patients, 56% were males (n = 122) and 44% (n = 96) females. The median age of these patients at initial diagnosis was to 63 years (range 31–86 years). The median age at tumor board presentation was 65 years (range 33–87 years), and this refers to all MMTBs in 2023.

The most common diagnosis was MM (72%, n = 209), followed by monoclonal gammopathy of undetermined significance (16%, n = 45), amyloidosis (8%, n = 23), smoldering multiple myeloma (6%, n = 17), plasmacytoma (5%, n = 13), and a different diagnosis was made for 6 patients (2%). For some patients, multiple diagnoses overlapped. Details regarding disease characteristics are summarized in Table 2.

Most patients presented at initial diagnosis (73%, n = 211), 3% without disease (n = 8), 11% at first relapse (n = 31), 4% at the second relapse (n = 13), 4% at the third relapse (n = 12), and 15 patients had already had more than three relapses (5%). In addition, it was determined whether the MMTBs took place specifically before or after high-dose chemotherapy with autologous stem cell therapy (HDCT/ASCT) or before or after CAR-T cell therapy. Twenty-two percent (n = 64) of the MMTBs were held before HDCT with ASCT, while 11% (n = 31) were held after HDCT with ASCT. Before CAR-T cell therapies, 6% (n = 18) of MMTBs occurred. Only 1% (n = 3) of MMTBs happened after CAR-T cell therapy. In total, 60% (n = 174) of MMTBs took place at a different treatment time (e.g., at progression and before treatment modification).

### 3.3. Adherence to Diagnostics and Therapy Recommendations

Table 3 shows that, according to our adherence definition, 86% (n = 251) of MMTB recommendations were followed. Of these, 84% (n = 244) were completely adhered to and 2% (n = 7) incompletely. In 4% (n = 11) of cases, it was not possible to determine whether the MMTB recommendations had been followed due to a lack of documentation. A total of 10% of all measures deviated from the original MMTB recommendations. In absolute figures, 28 MMTB decisions that were not adhered to. All non-adherent MMTB recommendations were justified and plausible. The main reason for deviations was patient decision (n = 21, 75%). Other reasons were changes in the situation that made the MMTB recommendation no longer medically justifiable in the further course (n = 4, 14%), cost reimbursements not received (n = 2, 7%), and death of the patient (n = 1, 4%).

### 3.4. Adherence to Proposed Trial Participation

In total, 36% (n = 104) of MMTBs included recommendations for study inclusion. Study inclusion occurred in only 32% (n = 33) of cases, and 68% of cases did not lead to study participation. In absolute numbers, there were 71 recommendations for study participation that were not adhered to. In 41% (n = 29) of the cases, a reason could be found. The reasons were patient decision (e.g., desire for treatment close to home), failure to meet inclusion criteria, studies not yet open, rejected cost coverage, and changes in the situation that made the study recommendation no longer medically justifiable in the further course of treatment (Table 4).

In individual patients who were presented to the MMTB several times, participation in the same study was repeatedly proposed. Each MMTB presentation was evaluated as a single case.

## 4. Discussion

The current study examined adherence rates to recommendations formulated at MMTBs held at the University Hospital of Bern over a period of one year. To assess adherence, we cross-compared recommendations given for each individual MMTB case with diagnostic and therapeutic procedures ultimately implemented.

Overall, patient basal characteristics were comparable with previous MM studies, except for median age at diagnosis, which was 4 years younger [7,10]. Typically, the incidence of the disease was slightly higher in men [2,7,10,25].

While earlier studies in MM reported MTB adherence rates around 93–94%, our study showed a slightly lower degree of adherence at 86% (n = 251) [7,10]. Encouragingly, all deviations regarding diagnostics and therapy could be justified. Previous studies in other tumor entities showed high heterogeneity in adherence rates to MTBs recommendations, ranging from 58% to 92%. The reported adherence was lowest for gastrointestinal cancers (58–87%) and highest for head and neck (78–92%), neuro-oncological and gynecological tumors (80%), hepatocellular carcinoma (85%) and lung tumors (90%) [11,12,20,23,26,27,28,29,30,31]. Braulke et al. reviewed adherence rates to MTBs for multiple tumor entities, reporting an overall adherence rate of 92%, which reached 100% for hematological malignancies [21]. However, a direct cross-comparison with previous studies is challenging due to high heterogeneity in methodology. For future studies, the use of uniform adherence definition and assessment would be of relevance [20].

In line with previous reports, the most frequent reason for non-adherence was patient decision [7,10]. Previous studies also reported that patient preferences are one of the main reasons for not adhering to MTB recommendations, underlining the relevance of patient-centered clinical decisions [11,12,20,21,26,27,28,29]. Some studies also identified the dynamic clinical condition as a relevant reason for deviations [11,12,20,28]. The study by Cao et al. emphasized that an optimal adherence rate might not be necessarily 100% [28]. Unfortunately, comprehensive information on patient clinical condition, comorbidities, and preferences is frequently not available during MTBs [11]. Adherence rates might be improved by addressing patient preferences during MTBs or through direct patient participation in selected MTBs [11,20,21,27]. Future studies might investigate reasons that patients made decisions that differed from the recommendations. Fear of side effects was the most common reason in the study by Rangabashyam et al. [27]. In our study, reasons for patient refusal were only documented in selected cases. Additionally, optimization of communication between physicians and patients is essential for treatment acceptance [27].

A much larger non-adherence rate was observed for recommendations concerning clinical trial enrolment. In our study, trial enrolment occurred only in one third of the cases. Previous studies reported higher trial inclusion rates following MTB recommendations. For instance, Engelhardt et al. reported an inclusion rate of 53.2%. In this study, main reasons for non-inclusion were unmet inclusion criteria or patient decisions [10]. In our cohort, reasons for non-adherence were documented in 41% of the cases and included patient decision, failure to fulfil inclusion criteria, delays in recruitment, rejected cost claims and changing clinical condition. The study by Kuroki et al., which reviewed gynecological MTBs, showed comparable results. In total, 30% of MTBs identified patients who were suitable for studies, and only 24% of these were ultimately included in studies [13]. In the study by Dapper et al., which examined multi-entity MTBs, a recommendation for participation in a study was formulated in 7% of MTBs. Of these, 38% of patients were finally enrolled in the study. Common reasons for non-enrolment were unfulfilled inclusion criteria, patient preference, or external treatment, remaining unclarified in approximately half of the cases [19].

Clinical trials are essential for developing new cancer therapies and might improve certain clinical outcomes [14,32,33]. However, a recent meta-analysis by Iskander et al. questioned this benefit for trial participants, showing that after adjusting for confounders and biases, no survival benefit was observed [16]. Moreover, only a minority of all cancer patients are eligible for and participate in clinical trials [13,14,18,19,32,34]. This was also confirmed in our study. Nipp et al. showed that logistical issues, non-fulfilled inclusion criteria, lack of resources, difficulties for patients and treating physicians in dealing with the uncertainty associated with participation in clinical studies, and difficulties in assessing the risk-benefit ratio are common [32]. Michaels et al. concluded that, despite extensive literature analyzing these barriers, there is a lack of high-quality evidence to guide efforts to increase participation in clinical trials for cancer care [18]. A better logistic support and higher availability of resources would possibly minimize this “patient-independent” lack of adherence.

According to the study by Dapper et al., MTBs play a central role in promoting clinical study participation [19]. Further studies point to similar results [7,10,13,14]. In our study, the high number of cases in which no documentation regarding study participation was found raises the question of whether all recommendations for study participation were discussed with the patients. Kuroki et al. suggested that measures to facilitate these discussions with patients after MTBs would lead to improved study participation [13].

Regular analyses of adherence rates to MTB recommendations and reasons for non-compliance might improve MTB’s quality and efficiency. Our study highlighted the relevance of comprehensive medical documentation for quality assessments. Specifically, for patients treated with HDCT/ASCT, follow-up data is automatically collected in a structured documentation software, which relevantly reduced the number of non-evaluable cases. For non-transplant patients treated outside our institution, data availability from follow-up reports was limiting in selected cases. This underlines the relevance of systematic follow-up documentation to enable quality assessments. Mandatory feedback on implementation of MTB recommendations, along with documenting non-adherence could facilitate future evaluations [10].

Some limitations of the current work are the retrospective and single-center study design and a relatively short observation period. Furthermore, our study mainly reviewed adherence. Future studies might additionally analyze whether formulated recommendations are in line with guidelines or lead to improved trial enrolment. Another unanswered question is whether patient- or tumor-specific factors additionally impact adherence. Braulke et al. reported no association between adherence to recommendations and patient age, gender or distance of residence from the treating center [21]. Soon et al. found an association between non-adherence to recommendations and advanced age or tumor stage [26]. Furthermore, the design of our study does not allow usto assess whether adherence to MTBs correlates with better clinical outcomes, as suggested by previous studies [31].

## 5. Conclusions

Our study showed high levels of adherence to MTBs recommendations for diagnostic procedures and therapy decisions. However, only approximately one third of recommendations concerning clinical trial enrolment were implemented in clinical practice. Frequent reason for non-adherence was patient preferences. Our work highlights the relevance of regular assessments of adherence rates to MTB recommendations as a quality control and suggests that considering patient preferences in MTB discussions might minimize deviations.

## Figures and Tables

**Table 1:** General information and recommendations of the multidisciplinary myeloma tumor boards.

Parameter	n	% ^1^
Evaluation period	January 2023–December 2023	
# Tumor board cases	290	100%
Median # TBs per week ^2^, (range)	6 (1–15)	
Median # TBs per patient ^2^, (range)	1 (1–4)	
TBs per patient (number) 1, 2, 3, 4	162, 43, 10, 3	74%, 20%, 5%, 1%
TB Recommendations ^3^		
Additional diagnostics required ^4^	77	27%
Chemotherapy/Immunotherapy	108	37%
Radiotherapy	32	11%
Maintenance therapy	29	10%
HDCT/ASCT	67	23%
Tandem-ASCT	10	3%
CAR-T-cell therapy	18	6%
Bispecific antibody	7	2%
Watch-and-wait	59	20%
Other therapy recommendations	3	1%
Study participation	104	36%

n sample size, # number, TB tumor board, HDCT/ASCT high-dose chemotherapy/autologous stem cell transplantation, CAR-T-cell therapy chimeric antigen receptor T-cell therapy; ^1^ Percentage of all TBs (290); ^2^ During evaluation period; ^3^ Most TBs had multiple recommendations.; ^4^ In 16 (6%) TBs, further diagnostic clarification was recommended, and no therapy recommendation was given. 61 (21%) TBs included both therapy and recommendations for further diagnostics.

**Table 2:** Patient basal characteristics.

**Patient characteristics**	**n**	**%**
# Patients	218	100%
Males, females	122, 96	56%, 44%
Median age at diagnosis, (range)	63 (31–86)	
Median age at TB discussion, (range) ^1^	65 (33–87)	
**Disease characteristics**	**n**	**% ^2^**
Diagnoses ^3^		
MM	209	72%
SMM	17	6%
Amyloidosis	23	8%
Plasmacytoma	13	5%
MGUS	45	16%
Other ^4^	6	2%
Disease-specific data		
IgG	159	55%
IgA	58	20%
IgM	5	2%
IgD	1	0%
LC-only	49	17%
Other ^5^	18	6%
Kappa	164	57%
lambda	108	37%
Other ^5^	18	6%
(R)-ISS		
I	29	10%
II	67	23%
III	40	14%
NA	154	53%
Disease status		
No disease ^6^	8	3%
Initial diagnosis	211	73%
1. recurrence	31	11%
2. recurrence	13	4%
3. recurrence	12	4%
>3. recurrence	15	5%
Before HDCT/ASCT	64	22%
After HDCT/ASCT	31	11%
Before CAR-T	18	6%
After CAR-T	3	1%
Other treatment status	174	60%

n sample size, # number, TB tumor board, MM multiple myeloma, SMM smoldering MM, MGUS monoclonal gammopathy of uncertain significance, LC light-chain, (R-)ISS (revised) international staging system, NA not assessed; ^1^ of all Tbs in 2023; ^2^ Percentage of all TB cases (290); ^3^ Some patients had multiple diagnoses.; ^4^ Includes no or other diagnoses; ^5^ Includes asecretory/biclonal and none; ^6^ The suspicion of a recurrence or disease was not confirmed.

**Table 3:** Adherence to diagnostics and therapy recommendations of the multidisciplinary myeloma tumor board.

Parameter	n	%
Adherence ^1^		
Adherent	251	86%
Completely adherent	244	84%
Incompletely adherent ^2^	7	2%
Not adherent	28	10%
Unknown ^3^	11	4%

n sample size; ^1^ Recommendations for trial participation were not included in this analysis; ^2^ Therapy recommendations were adhered to, but suggested diagnostics were not carried out or it is not known whether the diagnostics were carried out; ^3^ not evaluable due to missing Follow-up information.

**Table 4:** Adherence to trial participation proposed by the multidisciplinary myeloma tumor board.

Parameter	n	%
# Tumor boards with proposed trial participation	104	36%
Adherence		
Adherent	33	32%
Not adherent	71	68%

*n* sample size, *#* number.

## Data Availability

No data supporting the reported results are deposited elsewhere.
